# Effect of Water Absorption on Electric Properties of Temperature-Resistant Polymers

**DOI:** 10.3390/polym16040521

**Published:** 2024-02-15

**Authors:** Kaito Watanabe, Masahiro Kaneko, Xianzhu Zhong, Kenji Takada, Tatsuo Kaneko, Mika Kawai, Tetsu Mitsumata

**Affiliations:** 1 Graduate School of Science and Technology, Niigata University, Niigata 950-2181, Japan; 2 Graduate School of Advanced Science and Technology, Japan Advanced Institute of Science and Technology, 1-1 Asahidai, Nomi 923-1292, Japan; 3 School of Chemical and Material Engineering, Jiangnan University, Wuxi 214266, China

**Keywords:** heat-resistant polymer, polyimide, poly(ethylene terephthalate), water absorption, electric conductivity, electric resistivity, dielectric constant

## Abstract

The effects of water absorption on the electric resistivity and dielectric constant of polyimide (PI) and poly(ethylene terephthalate) (PET) were investigated, and the mechanism of deterioration in electrical insulation properties was discussed. The polyimides are poly(oxydianiline pyromellitimide) (PMDA–ODA) and poly(para-phenylene diamine biphenyltetracarboxydiimide) (BPDA–PDA). These polymer films were immersed in pure water for various immersion times at room temperature, and the water absorption ratio was evaluated. The electric resistance for these films was measured at room temperature using a high-resistance meter, and the dielectric constant at room temperature was measured using an LCR meter in a frequency range of 200 kHz to 2 MHz. The absorption ratios at equilibrium absorption for PMDA–ODA, BPDA–PDA, and PET were 2.7, 2.5, and 0.5%, respectively. The critical volume fraction of the percolation threshold of electric conductivity due to water absorption was 0.034 for both PMDA–ODA and BPDA–PDA. On the other hand, PET did not show a significant decrease in the resistivity. For both PIs and PET, the dielectric constant observed could be explained by a series model of the respective capacitances of pure water and polymer. Actually, the resistivity of samples cut from the edges of the film after water absorption was almost the same value as that in the dry state. These results suggest that the absorbed water molecules are not uniformly dispersed in the film but are localized at the edges of the film even after the absorption equilibrium has been reached.

## 1. Introduction

Heat-resistant polymers have been studied for a long time [1] and have been widely used as various industrial products in recent years [2,3,4,5,6], e.g., heat-resistant tableware, automotive lighting components, copier gears, organic light emitting diode (OLED) displays, etc. Polyamide is a typical heat-resistant polymer; for example, nylon six has a melting point of 215 °C [7], NomexTM has a melting point of over 520 °C [7], and aramid nanofiber separators have a 5 wt% decomposition temperature of 447 °C [8]. In particular, aromatic polyimides with aromatic rings directly linked to imide bonds demonstrate high heat resistance, excellent dielectric insulation properties, dimensional stability, solvent resistance, thermal stability, and high mechanical strength. They are widely used for adhesives, dielectrics, photoresists, non-linear optical materials, and separation membranes that can be used at high temperatures [9]. Thermoplastic aromatic polyimides are industrially useful, showing a glass transition temperature of 250 °C and a melting point of 388 °C [10].

Aromatic polyimides also have a great advantage in electrical insulating properties, as well as epoxy resin [11,12,13,14,15,16,17,18]. That is, aromatic polyimides are electric insulators that can be used at high temperatures. According to the literature, the dielectric constant and electric resistivity for conventional aromatic polyimides are distributed in approximately 3.0–4.0 [11,12,13,14] and 10^15^–10^16^ Ωcm [15,16,17,18], respectively. Recently, the remarkable progress of electronic devices capable of high-speed communication has further increased the demand for electronic substrates with lower dielectric constants and higher electrical insulation properties.

However, aromatic polyimides have the disadvantage that the excellent electric property falls significantly due to humidity, i.e., water absorption [10,19,20]. Generally, not only polyimides but also most polymers absorb moisture from the atmosphere, and their water absorbency depends on the chemical structure of the polymer. For example, the water absorption of the aromatic polyimides mentioned above is relatively high at 2.51% [11], and other examples are 10% for polybenzimidazole [21], 3.4% for CYCOM 977-2 epoxy resin [22], and 1.91% for 2,2-bis(4-cyanatophenyl)propane [23]. Conversely, the water absorptions of poly(ethylene terephthalate) and poly(phenylene sulfide) are relatively low at 1.1% [24] and 0.026% [25], respectively. In spite of that, the water absorption is, at most, a few percent, and the deterioration in electrical properties due to the water absorption is quite serious, as seen in the electric properties measured in various humidities [10,19,20,26]. It is because of this that the electric conductivity and dielectric constant of pure water are much higher than those of polymers. Actually, although it is not polyimide, the electric resistivity of ortho-cresol novolac epoxy resin, which is widely used as an electrical-insulating material, drops from 7.69 × 10^16^ Ωcm to 2.32 × 10^16^ Ωcm, with a water absorption of only 1.5% [27]. The electric resistivity of epoxy resin, a laminated structure consisting of alternating plies of 80 μm-thick mica paper and 50 μm-thick woven glass cloth, drops from 10^16^ Ωcm to 10^9^ Ωcm, with a water absorption of only 1% [28].

Understanding the water absorption process of polymers is very important to solve the problem of deterioration in the electrical properties mentioned above. The uptake of moisture or water absorption in polymeric films and the interaction between polymers and water have been under investigation for almost a century. For example, R. Buchhold et al. measured the change in infrared (IR) absorption spectra for PMDA–ODA prepared by spin coating with a thickness of 2.4 μm at various relative humidities to monitor intermolecular interactions using a method of attenuated total reflection (ATR-IR) [29]. Analysis of the sorption mechanism and the location of the sorption sites revealed that the water uptake into the polyimide film is caused by the sorption of water molecules into micropores inside the polymer. They described in the literature what exactly drives the uptake of moisture and how water uptake depends on morphology Also, S. Z. Li et al. investigated the nature and distribution of water molecules in polyimide (Kapton) films with various thicknesses by diffusion and deuteron (^2^H) nuclear magnetic resonance spectroscopy with complementary studies of IR absorption and differential scanning calorimetry. They reported that water exists in two distinct sites called γ_1_ and γ_2_, neither of which are strongly bound to the host polymer nor are they isotropically free. The water molecules in the γ_1_ site are distributed uniformly throughout the volume of the film, while those in the γ_2_ site (which are filled only at higher values of relative humidity) appear to be in small molecular clusters [30].

Thus, there is some information on the water absorption process and mechanism of polyimides, but it is still not fully understood. Also, it is empirically evident that water absorption leads to a significant decrease in the insulation properties of polymeric materials; however, a solution for its improvement has not been found yet. As a first step to solving this problem, it is necessary to elucidate the formation of conductive paths or polarization mechanisms caused by water absorption. In this study, the effect of water absorption on the electric properties of polyimide and poly(ethylene terephthalate), which are representative materials of heat-resistant polymers with different water absorbency, were investigated to elucidate the deterioration in insulation properties for these polymers.

## 2. Experimental Procedures

### 2.1. Samples

Kapton^®^ of poly(oxydianiline pyromellitimide) (PMDA–ODA) (Toray-Dupont Co., Ltd., Tokyo, Japan) and UPILEX^®^-S of poly(para-phenylene diamine biphenyltetracarboxydiimide) (BPDA–PDA) (Ube Co., Tokyo, Japan) were used as samples of polyimides. Lumirror^®^#125-S10 of poly(ethylene terephthalate) (PET) (Toray Industries, Inc., Tokyo, Japan) was used as a sample of engineering plastics with heat resistance and low water absorption. The thickness for all samples was 125 µm. Samples were square with a size of 40 × 40 mm for electric resistance measurements and 15 × 15 mm for dielectric constant measurements. These films were immersed in pure water for 3 days to remove water-soluble impurities before the absorption experiment and electrical measurements.

### 2.2. Water Absorption Measurements

PIs were dried in an oven at 150 °C under atmospheric pressure for 18 h, and PET was dried in a vacuum for 18 h at room temperature. After drying, the films were immersed in pure water at room temperature for a certain period of time. The weight of the films was measured by an electric balance (A&D Co., Ltd., GH-200, Tokyo, Japan) before and after immersing the films in pure water. The absorption ratio for the films *Q* was determined from the following equation,

(1) $$Q=\frac{W_{2}-W_{1}}{W_{1}}\times 100$$

Here, *W*_1_ and *W*_2_ are the weights of the film before and after immersion in pure water, respectively. The volume fraction of water in the films after immersion was also determined in the percolation analysis, which was calculated from the weight of the film before and after immersion in pure water using the densities of pure water, PMDA–ODA, BPDA–PDA, and PET with values of 1.00, 1.40, 1.47, and 1.40 g/cm^3^, respectively.

### 2.3. Electric Resistance Measurements

The electric resistance for the films was measured by the dc 2-terminals method using a high-resistance meter (Hioki E. E. Co., SM-7120, Ueda, Japan) and a parallel-plate electrode (Hioki E. E. Co., SME-8311, Ueda, Japan) at room temperature. The electrode has a guard electrode made of conductive rubber. The measurement using the parallel-plate electrode is a general method for polymer films satisfied with the standard [31,32] which corresponds to the international standard [33] The measurement was carried out in a chamber with controlled humidities (R.H. 9–21%). An electric potential of 100 V was applied in the direction of the film thickness, which corresponds to an electric field strength of 800 kV/m. The electric current in the present study ranged from 6.3 × 10^−14^ to 3.5 × 10^−12^ A, which is 100–10,000 fold higher than the maximum resolution of the high-resistance meter. The volume resistivity for the films, *ρ*_v_, was calculated from the following equation,

(2) $$R_{v}=\rho_{v}\frac{l}{S}$$

Here, *R_v_* is the resistance measured, *S* is the area of the electrode, and *l* is the thickness of the sample. It is well known that the influence of noise cannot be completely eliminated in resistivity measurements on materials with high resistivity in the order of 10^17^. The large noise observed here is not due to the type of high-resistance meter. In the present measurement, the integration time was set to be 320 ms, which is the maximum value for the high-resistance meter. We measured the time profile of electric resistivity three times for the same sample. Then, the average value of the resistivity from 150 s to 180 s was determined, and the three resulting averages were used to determine the mean value and error.

### 2.4. Capacitance Measurements

The capacitance for the films was measured by the ac two-terminal method using an LCR meter (Hioki E. E. Co., IM-3536, Ueda, Japan) at 25.0 °C in a frequency range from 200 kHz to 2 MHz. The film was sandwiched between electrodes with a diameter of 10 mm made of brass fabricated in a laboratory. An electric potential of 1.0 V was applied in the direction of the film thickness. The relative dielectric constant *ε_r_* for the films was calculated from the following equation,

(3) $$C=\varepsilon_{r}\varepsilon_{0}\frac{S}{l}$$

Here, *S* and *l* are the area and thickness of the sample, respectively. *C* is the capacitance measured and *ε*_0_ is the dielectric constant of vacuum (=8.854 × 10^−12^ F/m).

## 3. Results and Discussion

**Figure 1:**
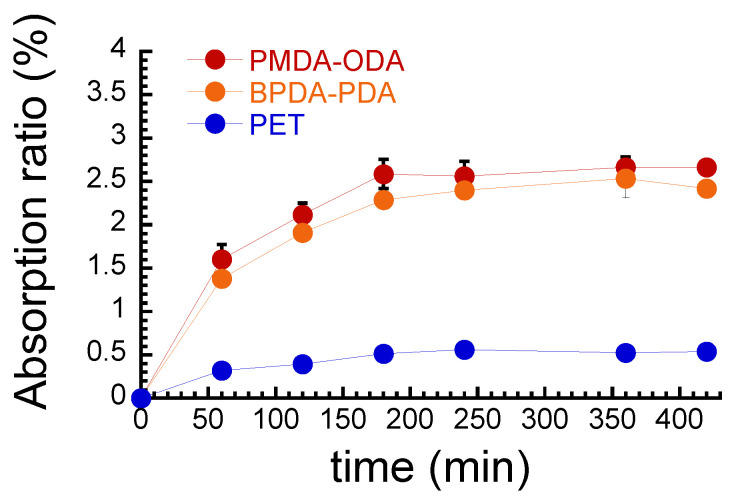
Relationship between water absorption ratio and absorption time for PIs and PET films.

Figure 1 shows the relationship between the water absorption ratio and absorption time for PIs and PET films. The absorption ratio for PMDA–ODA and BPDA–PDA gradually increased with time and became almost constant at 180 min. At the equilibrium, the absorption ratios for PMDA–ODA and BPDA–PDA were 2.7% and 2.5%, respectively. These values were in good agreement with the literature value of PMDA–ODA (=2.51%) [11]. The relatively high water absorption of PIs is due to the hydrogen bonding of the BI (benzimidazole imide) ring. Quan et al. reported that the ability to form intermolecular hydrogen bonding of the BI ring enhanced the possibility of hydrogen-bonded water molecule formations [34] and thus increased the water retention of corresponding materials [35,36,37]. It was also pointed out that the high water absorption in PI fabrications may cause the degradation of dielectric properties, dimensional changes, and delamination to restrict the commercial application of materials [35]. The absorption ratio for PET also gradually increased with time and became constant at 120 min. The absorption ratio for PET at the equilibrium was as low as 0.5%, which is slightly lower than the literature value of 1.1% [24].

**Figure 2:**
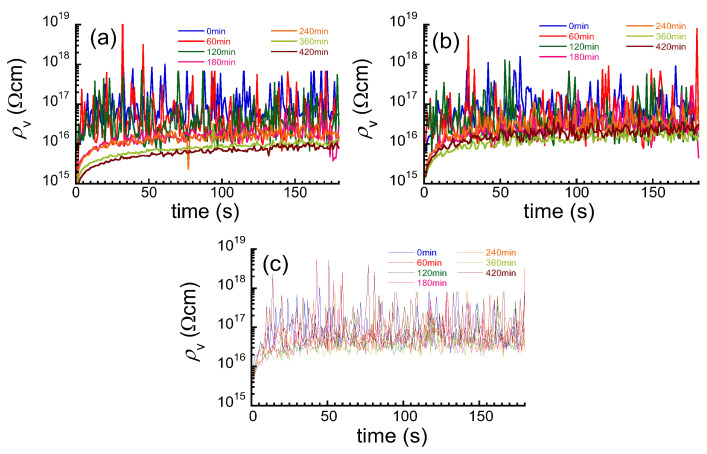
Time profiles of volume resistivity for PIs and PET films at various absorption times; (**a**) PMDA–ODA, (**b**) BPDA–PDA, (**c**) PET.

Figure 2a–c exhibit the time profiles of the volume resistivity for PMDA–ODA, BPDA–PDA, and PET films at various water absorption times, respectively. For all samples, the resistivity gradually increased with time due to the absorption current of charging [38]; then, it was almost saturated at 180 s. Although the high-resistance meter used in this study has a resolution of 0.1 fA, such extremely large noise with an order of 10^16^ was seen in the resistivity. The noise in the resistivity was large for all samples; however, the amplitude of the noise decreased with the water absorption. This is because the resistance was reduced to a range where it could be easily measured. To further improve the reliability of the current values measured, noise suppression should be employed. The average value was calculated using the data from 150 to 180 s when the value of volume resistivity was almost constant, although the noise was large.

**Figure 3:**
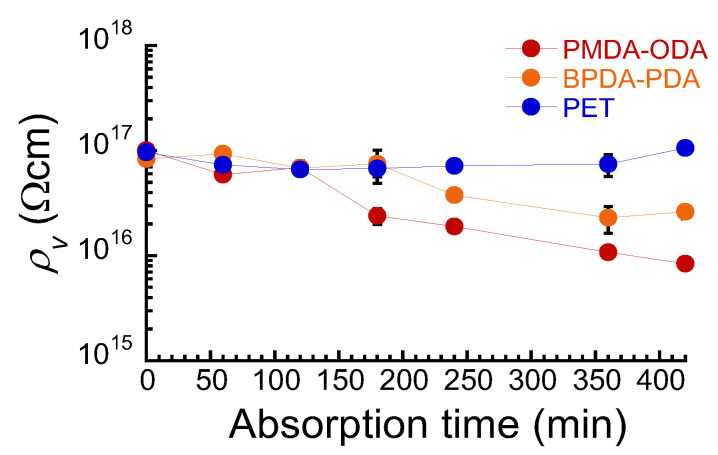
Volume resistivity at the equilibrium as a function of the absorption time for PIs and PET films.

Figure 3 shows the volume resistivity at the equilibrium as a function of the absorption time for PIs and PET films. In a dry state, the resistivity for PMDA–ODA was 1.01 × 10^17^ Ωcm, and that for BPDA–PDA was 8.32 × 10^16^ Ωcm. These values were an order of magnitude higher than the literature values of PMDA–ODA with 10^15^–10^16^ Ωcm [15,16,17,18]. It is well known that the electric resistivity for highly insulative polymers varies significantly depending on the type of measuring instrument and the water content of the sample. Furthermore, it depends on the humidity of the laboratory and the dryness of the measurement system. These experimental conditions and raw data, such as those in Figure 2, are not presented in many papers; therefore, it is not possible to directly compare these data with the experimental results. On the other hand, the resistivity for PET after drying was 9.59 × 10^16^ Ωcm. The resistivity for PIs decreased with the absorption time and became constant after approximately 360 min. The resistivity for PET also decreased with the absorption time and became constant after approximately 120 min. Thus, the resistivity was similar to the behavior of water absorption, suggesting that the decrease in the resistivity is caused by the water in which these films were absorbed. Gonon et al. observed that the electric resistivity decreased with an increase in water uptake, which is similar to our results. They explained that the decrease in the resistivity is due to a typical percolation phenomenon [28]. That is, the number of conducting channels in the epoxy resin increased when the water uptake increased. Paasi et al. also mentioned that the electric resistivity of polyester decreased when the relative humidity increased [39]. It was described in the report that the water absorbed into the material could contribute to free charge carriers and influence the carrier-trapping characteristics of the material. Although the PI film absorbed water even at absorption times below 100 min, there was no significant decrease in the resistivity, i.e., electrical conduction occurs after the polymer has absorbed a certain amount of water. This behavior can also be seen from the kinetic analysis of electric conductivity due to moisture absorption [40]. In the literature, Wang et al. described that the composite started showing conductivity after it absorbed approximately 50% of the maximum moisture. After this, conductivity increased quickly with further moisture absorption. The pattern of the increment of electrical conductivity suggests a diffusion process of moisture absorption. In our study, the water absorption with respect to the maximum water absorption was calculated to be 68% when the electric conductivity started to increase at an absorption time of 100 min. These values are almost identical, suggesting that the mechanism of the conductivity reduction due to moisture absorption is similar to that due to water absorption. To develop polymeric materials with high electrical insulation properties even after water absorption, it would be necessary to design materials so that water clusters do not percolate due to water absorption.

**Figure 4:**
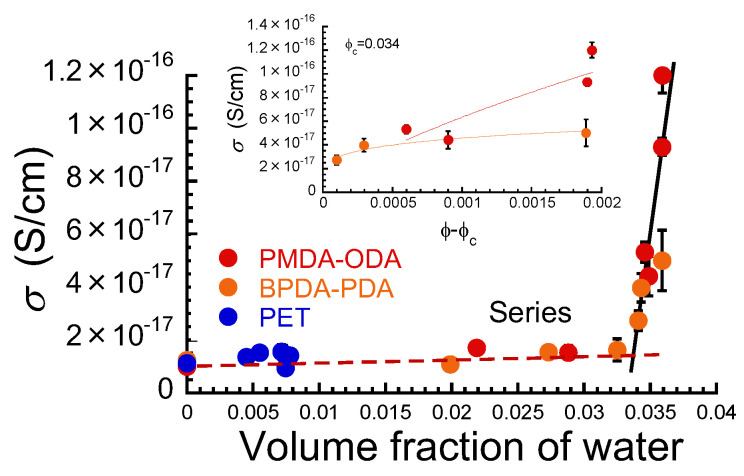
Relationship between electric conductivity and volume fraction of water for PIs and PET films. Broken line: series connection, Solid line: fitting line by a power law. Inset: Electric conductivity vs. *ϕ*–*ϕ_c_*.

Figure 4 demonstrates the relationship between electric conductivity at the equilibrium and the volume fraction of water absorbed in PIs and PET films. The electric conductivity *σ* is the reciprocal of the electric resistivity *ρ_v_*. The conductivity increased in proportion to the volume fraction of the water and showed a sharp increase at a volume fraction of around 0.034 for both PMDA–ODA and BPDA–PDA. The conductivity for PET also increased in proportion to the volume fraction; however, a sharp increase in the conductivity was not observed as in the case of PIs. At low volume fractions, the electric conductivity σ for the polymers after absorbing the water can be explained by the following equations when two resistances made of pure water and polymer are connected in parallel and series, respectively,

(4) $$\sigma =\frac{\sigma_{w}\sigma_{p}}{\phi_{w}\sigma_{p}+\left(1-\phi_{w}\right)\sigma_{w}}\;$$

(5) $$\sigma =\;\sigma_{w}\phi_{w}+\;\sigma_{p}\left(1-\phi_{w}\right)\;$$

Here, *σ_p_* and *σ_w_* are the electric conductivities of polymer and pure water, respectively. Values of 10^−17^ and 10^−7^ S/cm were substituted for *σ_p_* and *σ_w_*, respectively, which are of the same order as the experimental values. As shown in Figure 4, the broken line in the graph indicates the calculated values of the conductivity obtained from Equation (4) of the series connection of two resistors. For both PIs and PET, the experimental values were in good agreement with the calculated values. This strongly indicates that the electric current flows via both regions of the polymer and water absorbed in the films. The current flows through the region of least resistance between potential differences. If the absorbed water is unevenly distributed in the film, the current flows through the region where there are more water molecules. In addition, it can be seen that the ratio of the regions of polymer and water in the conduction path is exactly equal to that of the volume fraction of polymer and water. The conductivity calculated from Equation (5) of the parallel connection of two resistors overlapped the y-axis in this scale since the conductivity of the water is ~10^10^ folds higher than that of the polymers. This indicates that there is no path of an electric current that flows only via the water region or only via the polymer region.

At volume fractions above 0.034, the electric conductivity *σ* for PMDA–ODA and BPDA-PDA satisfied a power law expressed by the following equation,

(6) $$\sigma =\sigma_{0}{\left(\phi -\phi_{c}\right)}^{\beta }$$

where *σ*_0_ is the scaling factor, *ϕ_c_* is the critical volume fraction, and *β* is the critical exponent related to the dimensionality of the percolated network of electric conduction. The inset shows a relationship between the electric conductivity and *ϕ* − *ϕ_c_*, and it showed a power dependency as written in Equation (6). Therefore, the remarkable increase in the conductivity is considered to be a percolation behavior of electric conduction, even though the plots are not sufficient to be analyzed in detail. That is, the shortest path of electric conduction occurred at the critical volume fraction of *ϕ_c_* = 0.034. Such low values of the critical volume fraction have been reported for composites of epoxy resin and graphite nanoplatelets [41]. The critical exponents in Equation (6) for PMDA–ODA and BPDA–PDA were 0.7 and 0.2, respectively. The critical exponent of PMDA–ODA was higher than that of BPDA–PDA, suggesting that PMDA–ODA has many discontinuous paths that are not percolated. These values were much lower than those obtained from the universality of the critical exponent of electrical conduction (approximately 2) [42,43,44]. Alternatively, these values were much lower than the value of 1.725 [45] in three dimensions obtained by numerical calculations. However, such low values of the critical exponent for electrical conduction are not unusual, e.g., 0.91 for Aerosil 90 [46], 0.964 for SBA–15 [47], and 1.08 for hydrated yeast [48]. On the other hand, the critical exponent of the percolation probability is 0.4 [42], which was in good agreement with the critical exponent of electrical conduction obtained in this experiment. Therefore, this indicates that the percolation behavior of electric conduction observed in PI films is dominated by the percolation probability. In other words, it is considered that the electric conduction occurs through the largest clusters of water absorbed in the PIs films.

**Figure 5:**
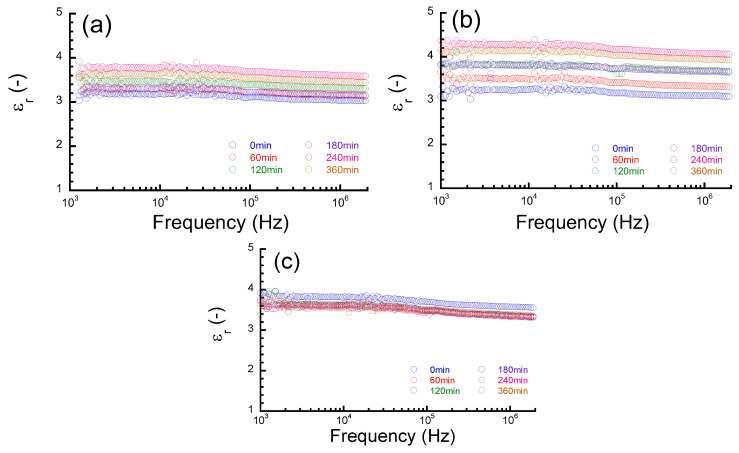
Frequency spectra for relative dielectric constant for PIs and PET films at various absorption times; (**a**) PMDA–ODA, (**b**) BPDA–PDA, (**c**) PET.

Figure 5a–c display the frequency spectra of the relative dielectric constant of PMDA–ODA, BPDA–PDA, and PET films, respectively. The dielectric constant for all films was independent of the frequency, i.e., these polymers do not show any dielectric relaxation or dielectric dispersion in this frequency range. Water molecules do not undergo dielectric relaxation or dielectric dispersion in this frequency range. Therefore, the observed dielectric constants should be constant in this frequency region. This means that the dielectric constant originates from the intrinsic polarization of constituent molecules of these polymers. That is, the dielectric constant is attributed to the intrinsic polarization of the constituent molecules of these polymers, i.e., their fast motion within a time scale over µs. It was observed for all films that the dielectric constant after water absorption was also independent of the frequency, although it increased with an increasing absorption time. This suggests that the dielectric constant is due to the summation of the electric polarizations of polymer and absorbed water and there is no molecular interaction between the polymer and absorbed water within the timescale of this frequency range.

**Figure 6:**
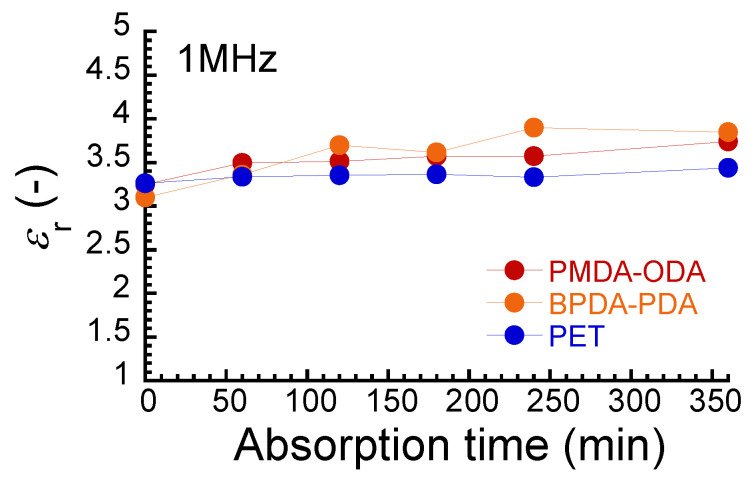
Relationship between relative dielectric constant at 1 MHz and absorption time for PIs and PET films.

Figure 6 shows the relationship between a relative dielectric constant at 1 MHz and absorption time for PIs and PET films. The dielectric constants for dried films of PMDA–ODA, BPDA–PDA, and PET were 3.2, 3.0, and 3.3, respectively. These values were in good agreement with the literature values of 2.94 for PI [14], 2.9–3.9 for PMDA–ODA [11,12,13,26], 3.1 for BPDA–PDA [12], and 3.4 for PET [49]. The dielectric constants for PMDA–ODA and BPDA–PDA increased with the absorption time and became constant at around 180 min. The increase in the dielectric constant is due to the water absorption similar to the resistivity. Kudus reported the high dielectric constant of a composite of epoxy resin and graphene nanoparticles (GNPs) [50]. It is because diamine-cured epoxies and GNPs have high water absorption due to the large number of hydroxyl groups. Thus, it is not rare that a high dielectric constant is caused by water molecules. The dielectric constant of water was approximately 25 times higher than that of the PIs, and only a small amount of water in the polymer caused the bulk dielectric constant to increase. In the present study, the electric polarization of these molecules was hypothesized to be invariable by absorbing water. Similar behavior was observed for the dielectric constant of Kapton measured at various humidities [26]. On the other hand, the dielectric constant for PET was independent of the absorption time, although the PET contained a small amount of water with an absorption ratio of 0.5%. Furthermore, the detection of water absorption was found to be more pronounced for the dielectric constant than for electrical resistivity.

**Figure 7:**
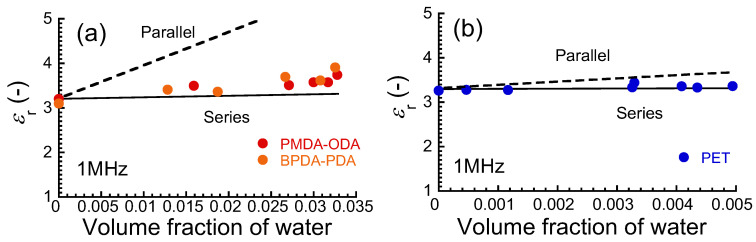
Relationship between relative dielectric constant at 1 MHz and volume fraction of water for (**a**) PIs and (**b**) PET films. Broken line: parallel connection, Solid line: series connection.

Figure 7a,b exhibits the relationship between the relative dielectric constant at 1 MHz and the volume fraction of water for PIs and PET films. When two capacitors made of pure water and polymer are connected in series, the observed dielectric constant *ε_r_* can be expressed by the following equation,

(7) $$\varepsilon_{r}=\frac{\varepsilon_{w}\varepsilon_{p}}{\phi_{w}\varepsilon_{p}+\left(1-\phi_{w}\right)\varepsilon_{w}}$$

Here, *ε_w_* and *ε_p_* are the dielectric constants of pure water and polymer, respectively. *ϕ_w_* is the volume fraction of pure water. When the two capacitors are connected in parallel, the observed dielectric constant *ε_r_* is expressed by the following equation,

(8) $$\varepsilon_{r}=\phi_{w}\varepsilon_{w}+\left(1-\phi_{w}\right)\varepsilon_{p}$$

The notation of symbols is the same as those in Equation (7). The dielectric constant for PIs increased in proportion to the volume fraction of the water. The solid and dashed lines in the graphs show the dielectric constants calculated in series and parallel connections, respectively, substituting the values of *ε_w_* = 80.1 and *ε_p_* = 3.2 for PI or 3.3 for PET. For both PIs and PET, the experimental values were close to the calculated values obtained in Equation (7) for the series connection. This strongly indicates that water molecules absorbed were not distributed homogeneously in these films; i.e., the molecules are localized in a limited space. However, it was also observed that the dielectric constant for PIs was slightly but clearly higher than the theoretical value for the series connection. This may be caused by the edges of the film because the distribution of water is relatively uniform at the edges of the film. 

**Figure 8:**
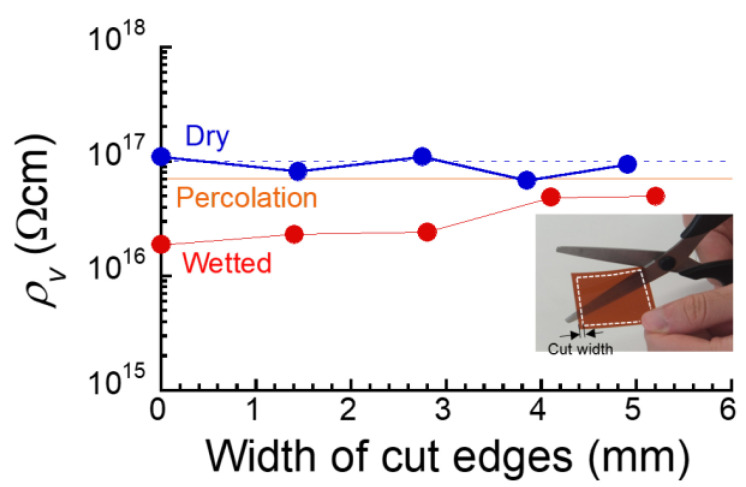
Relationship between volume resistivity and the width of cut edges for PMDA–ODA film. The resistivities of dried film and at the percolation threshold were indicated by the broken line and solid line, respectively. Inset: Photograph showing the cut width of the film.

To reveal the heterogeneous distribution of water molecules absorbed in the film, an additional measurement was performed on films with the edges cut off. The edges of four sides of a certain width were cut with scissors, and the electrical resistance of the remaining square of the film was measured. The time acquired for the measurement was around 20 min. Therefore, the increase in the resistivity observed is clearly not caused by drying the film due to water evaporation while the measurement.. Note that the water is absorbed not only from the edges of the film but also from the side. The cut-edge experiments for films after water absorption were performed in order to clear the distribution of water inside the films. An electric current flows through the area of least resistance between potential differences. Therefore, if the film is electrically non-uniform, the areas with low resistance can be detected. Figure 8 shows the relationship between volume resistivity and the width of the cut edges for the dry film of PMDA–ODA and the film immersed in pure water for 4 h. No apparent changes in the resistivity by cutting the edges were observed for a dried PMDA–ODA film without water absorption. In contrast, the resistivity for the film with an absorption time of 4 h clearly increased with the width of cut edges, and the resistivity at a width of 5.2 mm was very close to that at the percolation threshold, indicating that water molecules absorbed are localized at the four edges of the film, and the electrical conduction occurs predominantly via water molecules. As mentioned above, it should be noted that the water is absorbed not only from the edges of the film but also from the side. These results indicate that the film is electrically non-uniform, consisting of a high-water absorption area with low electric resistance (edges) and a low-water absorption area with high electric resistance (center). This finding was consistent with the model obtained from the results of the dielectric constant. Also, it was found that water penetrating through the top and bottom sides of the film was not the main cause of the deterioration in insulation properties.

**Figure 9:**
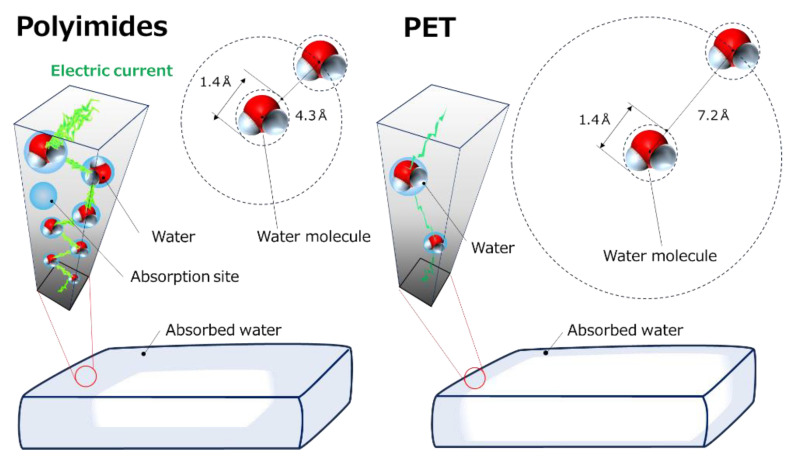
Schematic illustrations representing the mechanism for the significant deterioration in electric properties for PIs and PET films due to water absorption.

Figure 9 shows a schematic illustration of the mechanism of the decrease in electric resistivity and the increase in the dielectric constant due to water absorption for PIs and PET films. The illustration for PIs represents a state in which 92% of the absorption site is occupied by the water molecules (*ϕ* = 0.034); meanwhile, for PET, it represents a state where the maximum absorption is *ϕ* = 0.008. For both films, the volume fraction’s dependence on the dielectric constant suggests that water molecules are not uniformly absorbed in the films and are localized only at the surface of the films. This result coincides with the result obtained in the relationship between volume resistivity and the width of cut edges for PMDA–ODA film. Since PI and PET are hydrophobic, water is only absorbed at the polymer/water interface, where the concentration gradient is maximum. The concentration gradient should decrease rapidly inside the polymers; therefore, the diffusion of water would be greatly depressed. It was also found from the volume fraction dependence of electric conductivity for both films that the electric current flows through both domains of polymer and water absorbed. The fact that the electric resistance is a series connection of water and polymer is consistent with the finding from Figure 7, where the dielectric constant can be explained by a series connection of these capacitors. For PI films, a rapid increase in the electric current occurs via water molecules. No rapid increase in the electric current occurs for PET, and the ultrasmall current flows via a small amount of water absorbed. Assuming that the water absorbed in the films exists as a single molecule, the distance between the nearest-neighbor water molecules at the critical volume fraction, *r_ww_*, is expressed by the following equation,

(9) $$r_{ww}=\frac{r_{w}}{{\phi_{c}}^{\frac{1}{3}}}$$

Here, *r_w_* is the radius of the water molecule (=1.4 Å [51]), and *ϕ*_c_ is the critical volume fraction of water that equals 0.034 obtained in Figure 4. The hopping distances of ions contributing to the electric conduction in dry PI films have been reported to be 50–115 Å for Kapton-H [52,53,54] and 28 Å and 32 Å for PIs prepared from two different types of precursors [55], respectively. Water absorption reduced the hopping distance of ions from 1/27 to 1/12 of those in a dry state.

## 4. Conclusions

The effects of water absorption on the volume resistivity and dielectric constant for PIs and PET films were investigated. A serious deterioration in electrical insulation properties was observed for PI films at volume fractions above the percolation threshold. It was found that the absorbed water molecules were not uniformly distributed in the film but were localized at the surface of the film. This result was supported by an experiment in which the edges of the film were cut out. On the other hand, the electric conductivity for PET films increased in proportion to the volume fraction of the water; however, a serious deterioration in electrical insulation properties was not observed since the water absorption did not reach the percolation threshold. In order to ensure that the electrical insulating properties of such polymeric materials are not reduced remarkably by water absorption, it is important to design the material so that the percolation of electric conductivity does not occur at the top surface of the material, i.e., the absorption site of water molecules are not localized in polymeric films.

## Data Availability

Data are contained within the article.
